# Exendin-4 Exhibits Enhanced Anti-tumor Effects in Diabetic Mice

**DOI:** 10.1038/s41598-017-01952-5

**Published:** 2017-05-11

**Authors:** Lan He, Priscilla T. Y. Law, Chun Kwok Wong, Juliana C. N. Chan, Paul K. S. Chan

**Affiliations:** 1 Department of Microbiology, The Chinese University of Hong Kong, Prince of Wales Hospital, Shatin, New Territories Hong Kong SAR; 2 Department of Medicine and Therapeutics, The Chinese University of Hong Kong, Prince of Wales Hospital, Shatin, New Territories Hong Kong SAR; 3 Li Ka Shing Institute of Health Sciences, The Chinese University of Hong Kong, Prince of Wales Hospital, Shatin, New Territories Hong Kong SAR; 4 Department of Chemical Pathology, The Chinese University of Hong Kong, Prince of Wales Hospital, Shatin, New Territories Hong Kong SAR; 5 Institute of Chinese Medicine and State Key Laboratory of Phytochemistry and Plant Resources in West China, The Chinese University of Hong Kong, Prince of Wales Hospital, Shatin, New Territories Hong Kong SAR; 6 Hong Kong Institute of Diabetes and Obesity, The Chinese University of Hong Kong, Prince of Wales Hospital, Shatin, New Territories Hong Kong SAR

## Abstract

Type 2 diabetes (T2D) is associated with increased risk of cancers. In this connection, we previously demonstrated the promoting effect of diabetes on HPV-associated carcinogenesis using a xenograft model in db/db diabetic mice. The underlying mechanism of this observation might be partly contributed by dysregulated immune response in diabetes. In this study, we hypothesized that the impaired anti-tumor immune response in diabetic status could be modulated by exendin-4, a glucagon-like protein receptor agonist which exhibits anti-diabetic effects. We inoculated 10-week old db/db mice with 2 × 10^7^ CUP-1 cells (Human Papilloma Virus (HPV)-16 E7 transfected continuous cell line) subcutaneously underneath the scruff, and treated mice with high (30 nmol/kg) or low (10 nmol/kg) dose of exendin-4 for 13 days. Compared with control groups, exendin-4 suppressed subcutaneous tumor growth in a dose-dependent manner, accompanied by increased interferon (IFN)-γ secreting CD8^+^ cytotoxic T lymphocyte (CTL)/Foxp3^+^ regulatory T cell (Treg) ratio as well as Th1 proinflammatory cytokines IFN-γ and IL-2. Collectively, these findings suggested an anti-tumor effect of exendin-4 in diabetic conditions, which might be resulted from direct immunomodulation.

## Introduction

Type 2 diabetes (T2D) is associated with increased risk of all-site cancer (except prostate cancer)^1^. The diabetes–cancer link can be mediated by various hormonal (insulin, insulin-like growth factor1/IGF1, adipokines), immunological (inflammation), or metabolic (hyperglycemia) pathways which may be subject to modulation by treatments. In addition, obesity, infections and diabetes frequently co-exist to create a microenvironment characterized by inflammation and glucolipotoxicity which favors cancer growth^2^. However, the underlying mechanism remains poorly understood. One of the possible causes of this phenomenon is defects in host immunity, especially cell-mediated immunity. Db/db mice are overweight, develop severe insulin resistance, and serve as a model for type 2 diabetes and the metabolic syndrome^3^. In addition to the abnormal metabolic pathway, these mice are characterized by defective immune responses manifested by reduced antigen-specific T-cell proliferation and abnormalities in the number and function of dendritic cells, regulatory T cells and natural killer T cells^4, 5^. Yet, our knowledge on the correlation between diabetic status and host anti-tumor immune response is still very limited.

Glucagon-like peptide-1 (GLP-1) is a gut incretin hormone with anti-diabetic effects by augmenting post-prandial insulin secretion in a glucose-dependent manner, inhibiting glucagon secretion, inducing satiety, delaying gastric emptying and possibly promoting beta (β) cell proliferation^6^. Exendin-4 is a 39-amino acid peptide that shares approximately 53% homology with mammalian incretin GLP-1 and binds to and activates the mammalian GLP-1 receptor on pancreatic β cells. T2D is associated with reduced incretin effects, although the secretion of GLP-1 is not always decreased^7, 8^. Pharmacoepidemiological studies have shown that exendin-4 exerts anti-tumor effects in human pancreatic cancer cells and prostate cancer cells^9, 10^. Furthermore, GLP-1 receptor expression is widely detected in various cells and organs including kidney, lung, heart, hypothalamus, endothelial cells, neurons, astrocytes, microglia and pancreatic beta-cells as well as immune cells^11–14^. Our group has reported that T2D patients exhibited activated inflammatory responses of peripheral blood mononuclear cells (PBMC) which were attenuated by exendin-4 in *ex vivo* experiments^15^. These findings suggested that GLP-1 receptor activation might have additional roles other than glucose-lowering effects. Collectively, these data indicate the potential of exendin-4 for improving anti-tumor immune response which is critical for protection against cancer growth.

With this background, we hypothesized that exendin-4 may modulate tumor growth through improving anti-tumor immune response characterized by imbalance between cytotoxic and regulatory T cell immunity. To test this hypothesis, we developed a mouse model inoculated with HPV-carrying cancer cell lines and examined the *in vitro* and *in vivo* effects of exendin-4 on tumor growth and associated changes in the population of CD8^+^ CTL and Foxp3 Tregs and pro-inflammatory cytokines.

## Results

### Tumorigenicity of CUP-1 cancer cells in diabetic mice

**Figure 1:**
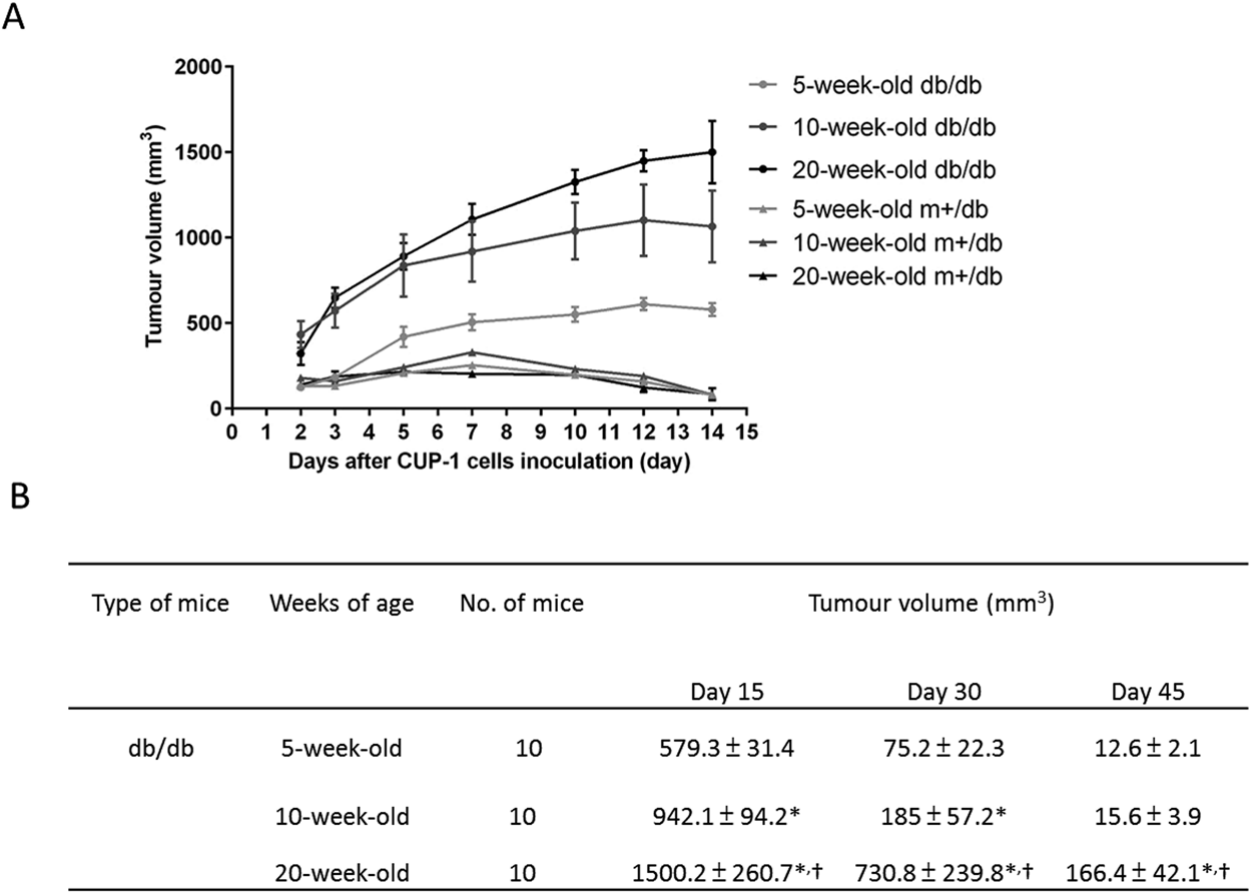
Tumorigenicity of CUP-1 tumor cell line *in vivo*. (**A**) The growth of CUP-1 cells, as tumor allografts in 5-, 10- and 20-week-old db/db and db/m+ control mice. After 2 × 10^7^ CUP-1 cells were injected subcutaneously into the scruff of mice, the short and long diameters of the tumors were measured weekly and tumor volumes (mm^3^) were calculated. Each point represents the mean ± S.E.M (n = 10). **p < 0.01, diabetic group compared to the control group. (**B**) The growth of 2 × 10^7^ CUP-1 allografts in 5-, 10- and 20-week-old db/db mice. *p < 0.05 compared to the corresponding 5-week-old group ^†^p < 0.05 compared to the corresponding 10-week-old group. Each point represents mean ± S.E.M (n = 10).

In order to study the tumorigenicity of CUP-1 tumor cells in diabetic microenvironment, db/db mice which developed overt hyperglycemia by 10 weeks and getting worse with age were used. We injected CUP-1 cells into 5-, 10- and 20-week-old db/db mice which represented early-, intermediate- and late-diabetic stage respectively and found that tumor size increased with age and severity of diabetes (Fig. 1A and B). Histological examination by haematoxylin-eosin (HE) staining confirmed presence of malignant cells in the tumor mass^16^. These results showed that the tumorigenicity of CUP-1 cells in db/db mice was further exacerbated by increasing age and severity of diabetes.

### Exendin-4 induced CUP-1 tumor regression

**Figure 2:**
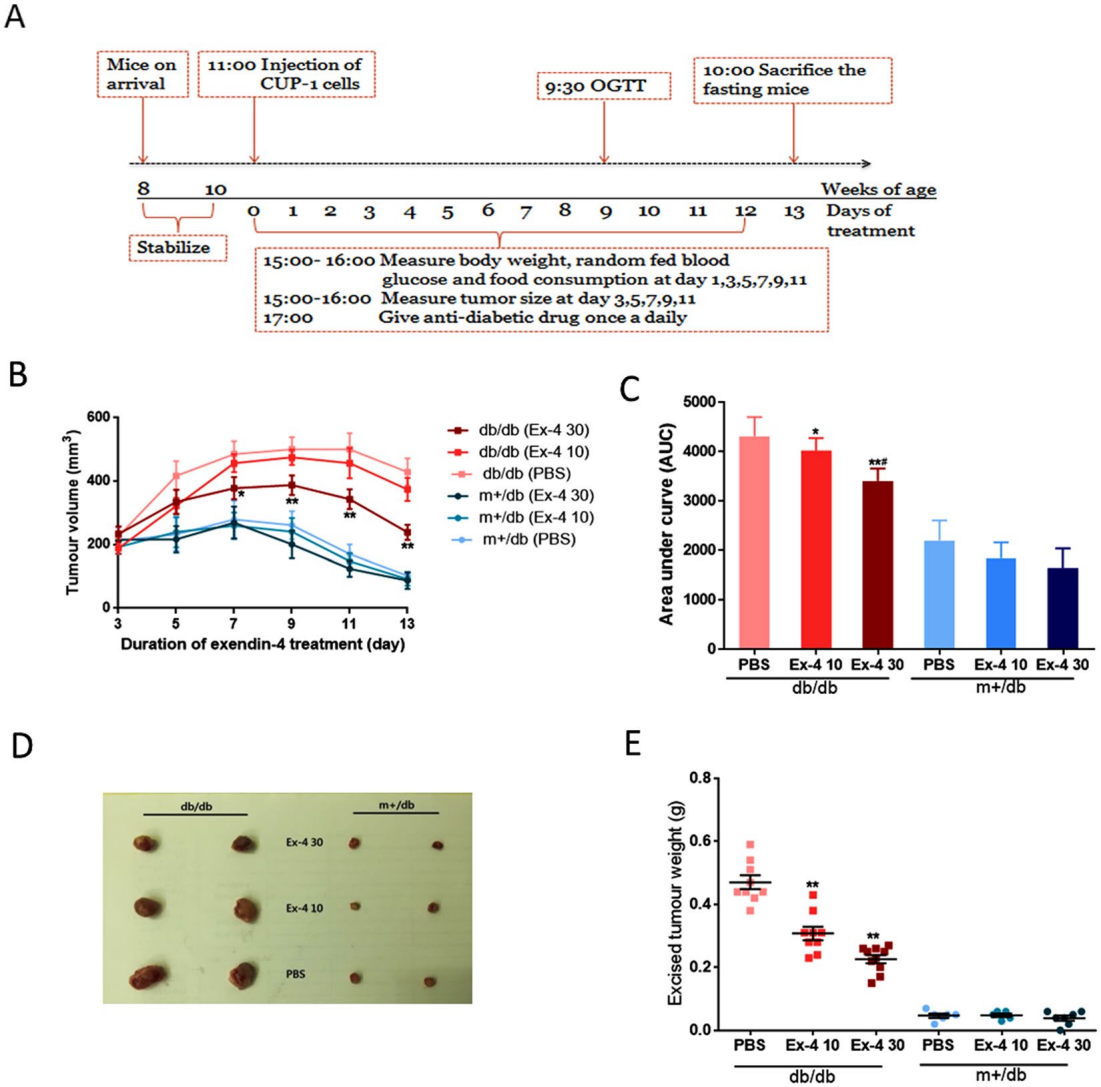
Effects of exendin-4 on tumor growth in db/db and db/m+ mice. (**A**) Experimental timeline of 13-day exendin-4 treatment in CUP-1 syngeneic db/db and db/m+ mice. Male db/db and db/m+ mice inoculated with 2 × 10^7^ CUP-1 cells were treated with exendin-4 intraperitoneally (10 or 30 nM/kg body weight) for 13 days (n = 10 per group). Tumors were measured every other day in the db/db and db/m+ mice (**B** and **C**). Mice were sacrificed and tumors were excised after 13 days of exendin-4 treatment. Mean excised tumor weight were compared between groups (**D** and **E**). Ex-4 30 and Ex-4 10: exendin-4 treatment at 30 and 10 nM/kg body weight. *p < 0.05, **p < 0.01, compared with PBS-treated group in the corresponding db/db or db/m+ group.

We then treated 10-week-old db/db mice bearing CUP-1 xenograft with high (30 nM/kg) or low (10 nM/kg) dose of exendin-4 and monitored the drug effects according to the regime as illustrated in Fig. 2A. Exendin-4 significantly suppressed CUP-1 xenograft growth in db/db mice in a dose-dependent manner (Fig. 2B). The high dose group had the lowest area under the curve (AUC) of tumor volume (3500.7 ± 210.3 mm^3^), followed by the low dose group (10 nM/kg) (4020.2 ± 251.2 mm^3^) and the PBS control group (4497.2 ± 197.4 mm^3^) in db/db mice (Fig. 2C).

After 13 days of exendin-4 treatment, all mice were sacrificed, and the CUP-1 tumors were excised. Fig. 2D shows a representative photograph of the excised CUP-1 tumors from different experimental groups. Amongst the db/db mice, the end-of-treatment tumor weight of the 30 nM/kg exendin-4-treated group was 54.2% lower than that of the PBS-treated group (0.22 ± 0.07 vs. 0.48 ± 0.12 g, P < 0.001, Fig. 2E). Although the AUC of tumor volume was similar between the 10 nM/kg exendin-4-treated and the PBS-treated group, the end-of-treatment tumor weight was significantly lower in the 10 nM/kg exendin-4 than the control group (0.31 ± 0.11 vs. 0.48 ± 0.12 g, P = 0.036).

The tumor-suppressive effect of exendin-4 was also observed in the non-diabetic db/m+ mice, albeit to a lesser extent (Fig. 2B). Exendin-4 treated db/m+ mice showed reduction in tumor volume between day 9 and day 11 of treatment. The AUC of tumor volume was 22% lower in db/m+ mice treated with 30 nM/kg exendin-4 than those treated with PBS (1645.1 ± 397.4 vs. 2200.0 ± 408.4 mm^3^, P = 0.041, Fig. 2C), although the excised tumor mass was similar between the 2 groups. Collectively, exendin-4 inhibited CUP-1 tumor growth in a dose-dependent manner in db/db mice and to a lesser extent in the non-diabetic (db/m+) mice.

### Exendin-4 attenuated hyperglycemia in db/db mice

**Figure 3:**
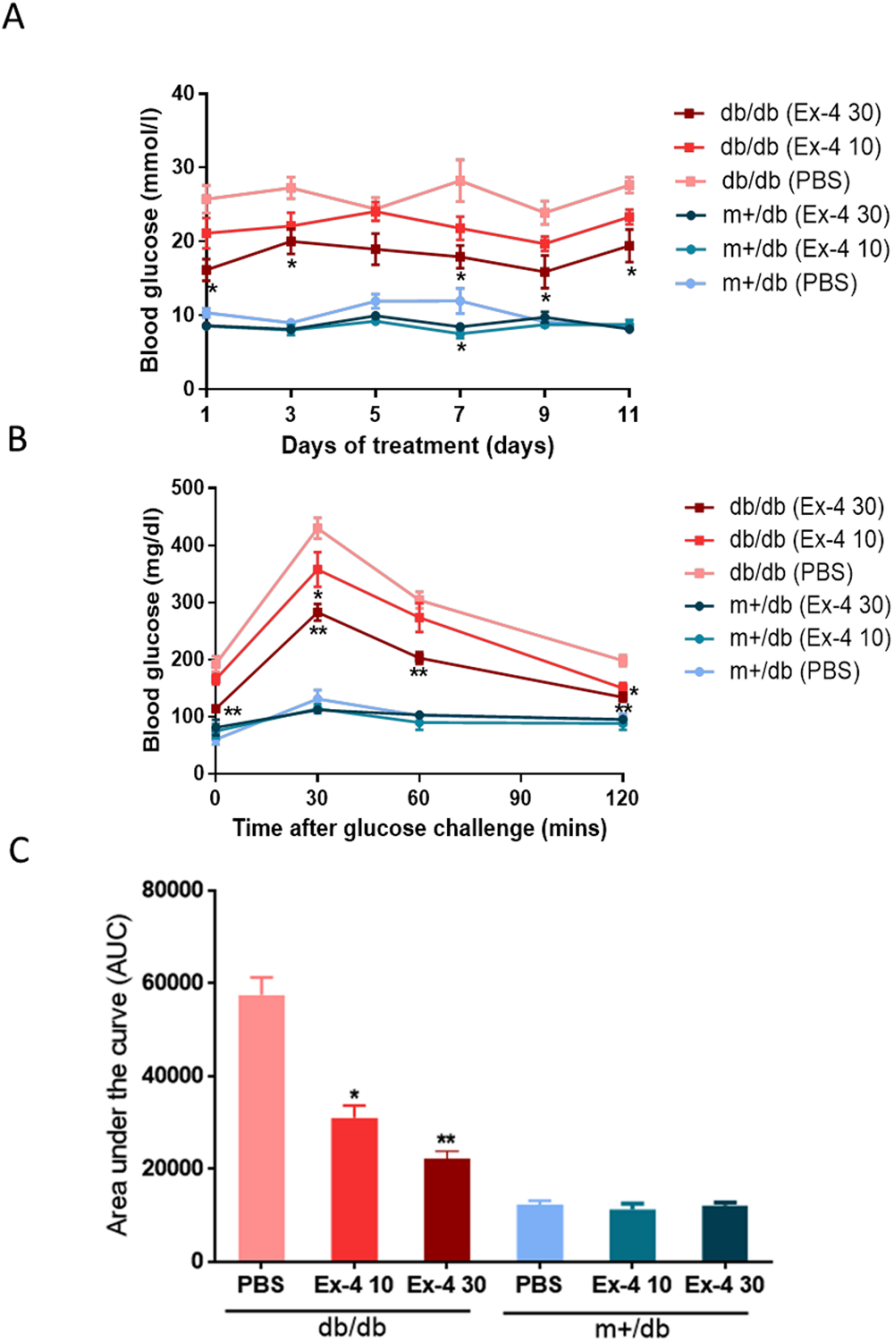
Effects of exendin-4 on blood glucose levels in CUP-1 allografted db/db and db/m+ mice. Male db/db and db/m+ mice inoculated with 2 × 10^7^ CUP-1 cells were treated with exendin-4 (10 or 30 nM/kg body weight, intraperitoneally) daily for 13 days (n = 10). Random blood glucose was measured at 9:30 am every other day (free access to food) (**A**), and OGTT was performed on day 9 at 9:30 am of exendin-4 treatment after overnight fasting (**B**). Glucose levels expressed as area under the curve (AUC) of glucose level during OGTT are shown in (**C**). Data are expressed as mean ± S.E.M. Ex-4 30 and Ex-4 10: exendin-4 treatment at 30 and 10 nM/kg body weight.

Compared to the PBS-treated group, daily treatment of 10 nM/kg and 30 nM/kg of exendin-4 reduced random blood glucose (RBG) in a dose dependent manner in the db/db mice (Fig. 3A), reaching significance for the 30 nM/kg dose. Based on the OGTT results performed on day 9, exendin-4-treated db/db groups had lower BG excursion than the PBS-treated db/db group. Exendin-4 treatment reduced BG concentration at all time points (0, 30, 60 and 120 minutes) (Fig. 3B) with the 30 nm/kg treated group having the lowest AUC of BG (22268.0 ± 4751.8), followed by the 10 nM/kg treated group (31060.7 ± 8257.8) compared to the PBS-treated (40407.8 ± 9390.8) group (Fig. 3C).

### Effects of exendin-4 on tumor-specific CD8^+^ T lymphocytes and tumor-specific Foxp3^+^CD4^+^CD25^+^Treg cells in tumor-bearing db/db mice

**Figure 4:**
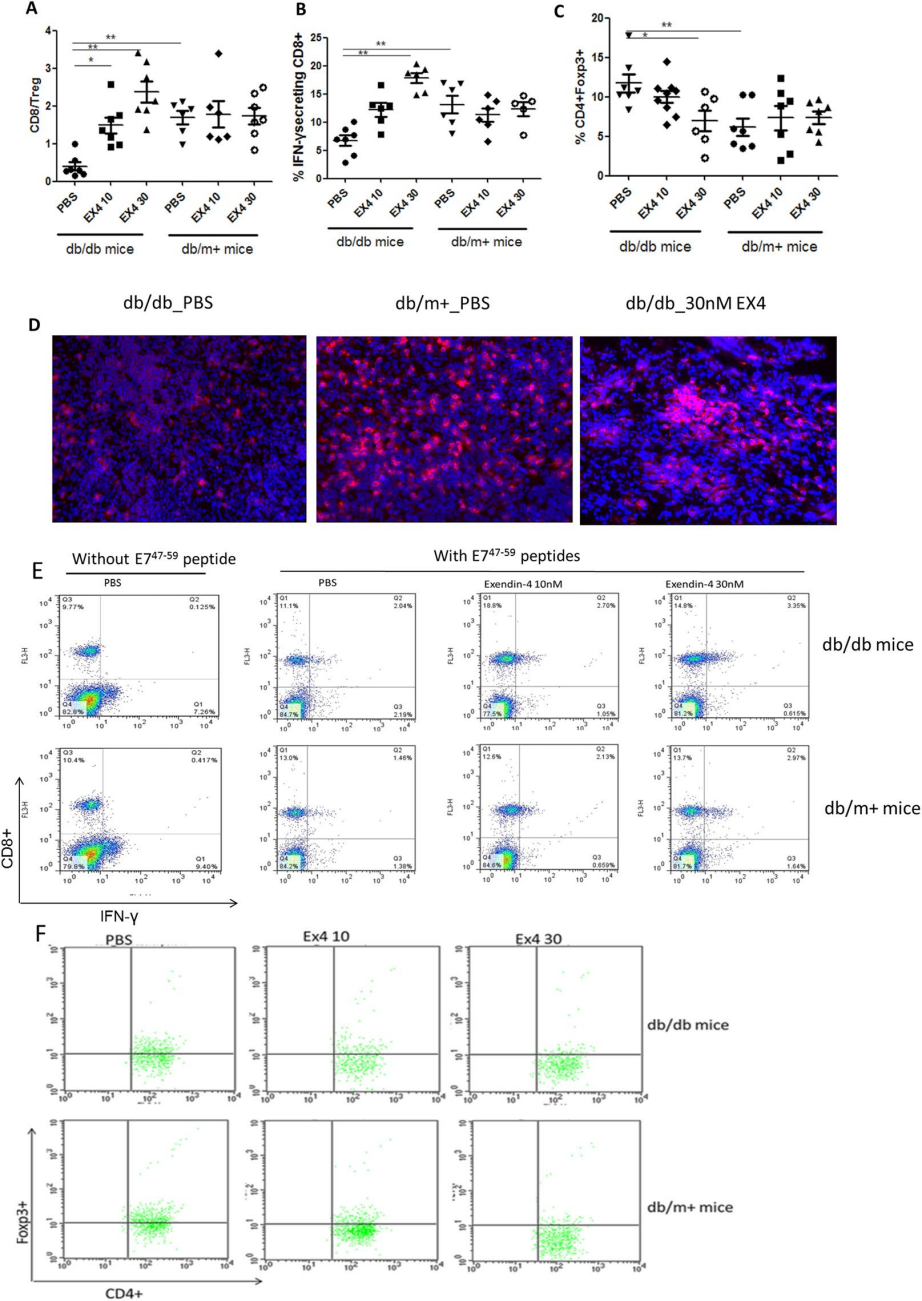
Anti-tumor T-cell response in mice treated with exendin-4. Male db/db and db/m+ mice inoculated with CUP-1 cells were treated with exendin-4 daily for 13 days. On day 13, splenocytes collected from sacrificed mice were cultured *in vitro* with 5 mg/ml HPV-16 E7^49–57^ peptides overnight, and stained for CD8^+^IFN-γ and CD4^+^ CD25^+^ Foxp3. (**A**) The CD8/Treg ratio was calculated by dividing the total number of IFN-γ+CD8^+^ T cells by the total number of CD4^+^Foxp3^+^ T cells in spleen. (**B**) Percentage of IFN-γ+CD8^+^ T cells was calculated using the total CD3^+^ T cells in spleen as denominator. (**C**) The percentage of Treg was calculated using the total CD4^+^ T cells in spleen as denominator. Circles represent individual mice (6–10 mice per group from two experiments). (**D**) Representative photographs of immunofluorescence staining for tumor-specific CD8^+^ T lymphocytes. Tumors obtained from mice treated with exendin-4 (30 nM) were stained for CD8^+^ T cells (red). Magnifications: ×200. (**E**) Representative flow cytometry measurement of IFN-γ secreting CD8^+^ T cells. (**F**) Representative flow cytometry measurement of CD4^+^CD25^+^FoxP3^+^Treg. Statistical significance was assessed by one-way ANOVA. *p < 0.05, **p < 0.01.

Having demonstrated that exendin-4 inhibited CUP-1 tumor growth in db/db mice, we investigated the effects of exendin-4 on host anti-tumor immune responses. We measured the number of tumor-specific CD8^+^ T lymphocytes and Foxp3^+^CD4^+^CD25^+^ Treg cells in tumor-bearing db/db mice. Using splenocytes harvested from the exendin-4 treated mice, we found increased E7-specific IFN-γ secreting CD8^+^ T cells (Fig. 4B and E) and decreased Foxp3^+^CD4^+^CD25^+^ Treg cells (Fig. 4C and F), which resulted in an increased CTL/Treg ratio (Fig. 4A) in a dose-dependent manner compared to that of PBS-treated controls. These responses were tumor-specific as no CD8^+^ T cell response was observed from splenocytes without E7^49–57^ peptide stimulation. Apart from inducing an increased CD8^+^ T cell to Treg ratio in db/db mice, immunofluorescence staining also demonstrated accumulation of tumor-infiltrating CD8^+^ T cells in the tumor tissue sections of db/db mice treated with exendin-4 (Fig. 4D).

### Effects of exendin-4 on tumor-specific CTL response

**Figure 5:**
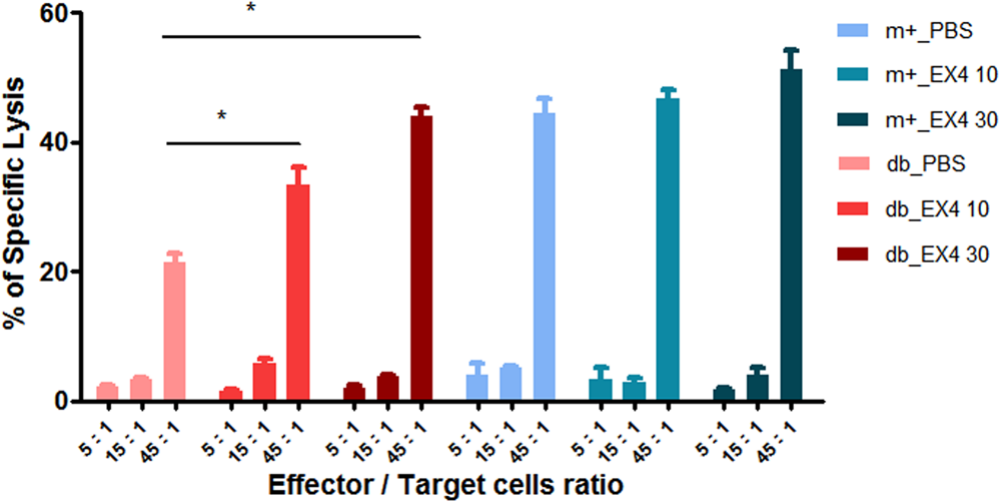
E7-specific CTL response induced by exendin-4. Male db/db and db/m+ mice inoculated with 2 × 10^7^ CUP-1 cells were treated with 10 nM/kg or 30 nM/kg of exendin-4 intraperitoneally daily for 13 days. After treatment, splenocytes were collected from sacrificed mice, subjected to 5 days of *in vitro* stimulation with HPV-16 E7^49–57^ peptides and used as effector cells. CTL activity was assessed by LDH assay using CUP-1 cells as target cells. Percentage of cytotoxicity (specific lysis) was calculated for cultures with effector:target ratios of 5:1, 15:1 and 45:1. Results are expressed as percentage cytotoxicity ± SEM. *p < 0.05.

Tumor-specific CTL response was examined by re-stimulating cultures of splenocytes harvested from exendin-4 treated and control mice with HPV-16 E7^49–57^ peptides. Viable effector cells were assessed for cytolytic activity against CUP-1 cells expressing HPV-16 E7. A dose-dependent increase in HPV-16 E7-specific cytotoxicity was observed in mice treated with exendin-4 but not in the control group. Treatment of exendin-4 elicited the highest level of HPV-16 E7-specific CTL (Fig. 5).

### Effects of exendin-4 on splenic cell culture supernatant concentration of cytokines

**Figure 6:**
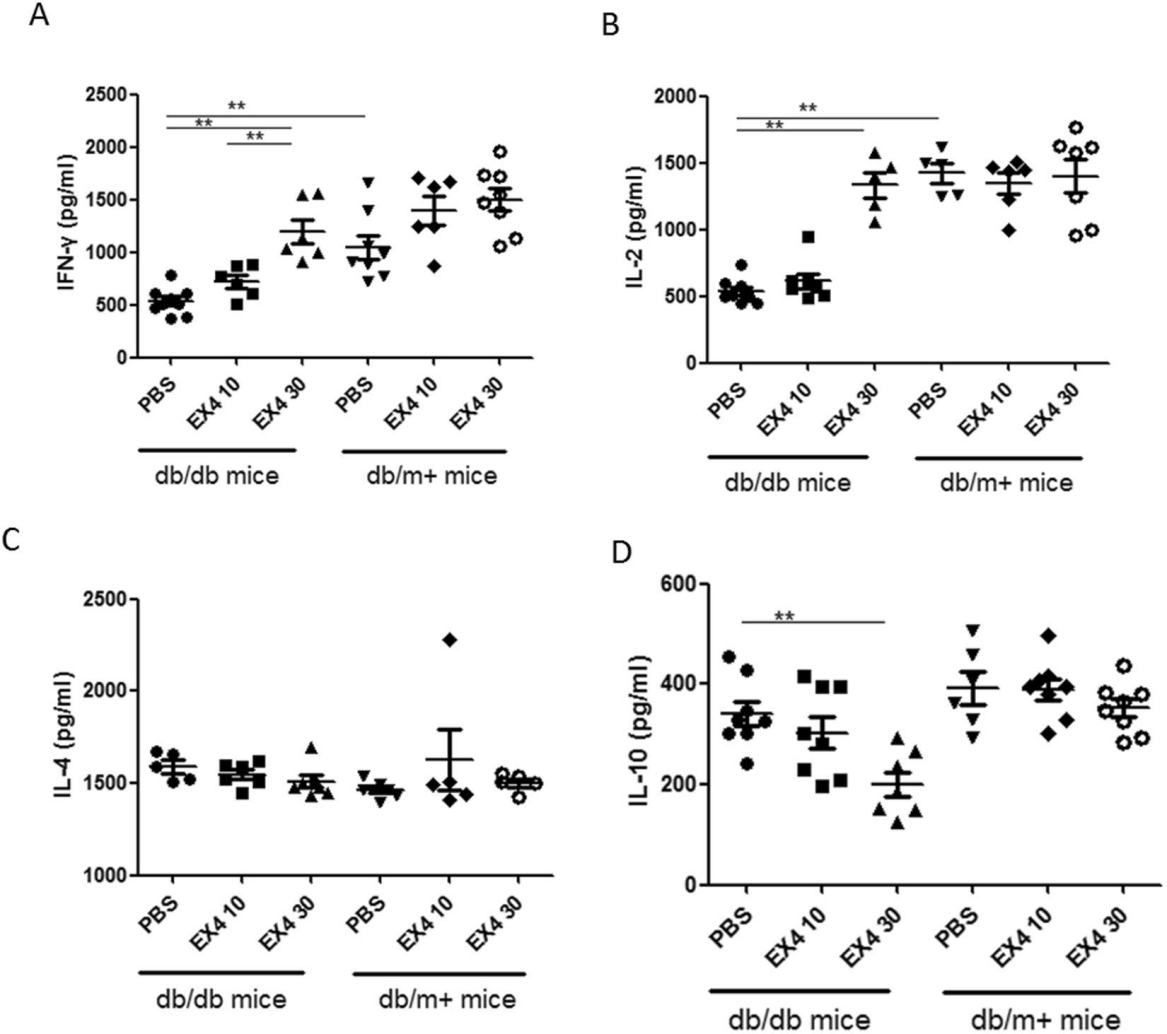
*In vitro* cytokine concentrations in supernatant of splenocyte culture collected from db/db and db/m+ mice treated with 10 nM/kg or 30 nM/kg of exendin-4. Mean concentrations of (**A**) IFN-γ, (**B**) IL-2, (**C**) IL-4 and (**D**) IL-10 measured from splenocyte culture supernatants at 48 h after phytohemagglutinin (PHA, 10 μg/mL) stimulation are shown. Data are expressed as mean ± SEM, n = 10. **p < 0.05 (n = 10 in each group).

To further investigate the anti-tumor effect of exendin-4 on immune cells, after 13 days of exendin-4 treatment, tumor-bearing db/db mice were sacrificed to obtain splenocytes to measure their capacity in secreting cytokines upon stimulation with HPV-16 E7^49–57^ peptides. As shown in Fig. 6, splenocytes from exendin-4 treated mice released significantly higher levels of inflammatory cytokines IFN-γ and IL-2 (Fig. 6A and B) and lower level of IL-10 (Fig. 6D) upon HPV-16 E7^49–57^ peptide stimulation than controls, while IL-4 levels remained unchanged (Fig. 6C).

## Discussion

In this study, we clearly demonstrated that exendin-4, an anti-diabetic drug, suppressed established solid tumors in db/db mice through mediating interferon (IFN)-γ secreting CD8^+^ cytotoxic T lymphocyte (CTL) and Forkhead Box P3 (Foxp3)^+^ regulatory T cells (Tregs). Interestingly, metformin, another well-known anti-diabetic drug, showed similar antitumor effect through inducing the number of CD8^+^ tumor-infiltrating lymphocytes (TILs) and protected them from apoptosis and exhaustion characterized by decreased production of IL-2, TNF-α, and IFN-γ^17^.

Diabetes has been shown to increase HPV pathogenicity in different diseases. For instance, patients with T2D are at increased risk of HPV-associated cancers, including cervical cancer and head and neck cancer^18–20^. Moreover, diabetic patients develop more extensive HPV-related genital warts and they are more prone to disease recurrence as compared to non-diabetic subjects^21^. In addition to epidemiological observations, our group has successfully shown that diabetes could act as a co-factor to promote HPV-driven tumorigenesis in db/db mice bearing CUP-1 (HPV-16 E7 transformed) xenograft^22^. We therefore tested whether exendin-4 could exert anti-tumor effect in these mice.

Significant inhibition of tumor development was observed in exendin-4 treated diabetic mice. The tumors were gradually and completely rejected with no reappearance after exendin-4 removal. A rechallenge with more than twice the original number of the same tumor cells did not yield any mass formation (data not shown), suggesting the generation of an immunologic memory response and the necessary involvement of T and/or B cells. Though several experimental studies have reported attenuated growth of colon cancer, breast cancer and prostate cancer upon exendin-4 treatment, all of these studies are based on *in vitro* experiments or immunodeficient mice^10, 23, 24^. There is little information about the role of exendin-4 on host anti-tumor immune function. Yet it has been reported that GLP-1 receptors are expressed in monocytes, macrophages, and lymphocytes, suggesting that GLP-1 mimetics might have potential to exert direct effects on host immune system.

Balance between Tregs and effector CD8^+^ T cells is an important determinant of host anti-tumor immune response in cancer biology. Foxp3 masters the regulatory pathway in Tregs development and function. The level of Foxp3 expressing CD4^+^ Tregs is a critical component in suppressing tumor immunity^25^. On the other hand, changes in CD8^+^ T lymphocytes with cytolytic activity (CTL) may have anti-tumor effects. As expected, our results demonstrated that intra-tumoral CD8^+^ CTL to Tregs ratio was activated in exendin-4 treated tumor-bearing diabetic mice. Consistent with the anti-tumor effects we observed in the tumor-bearing db/db diabetic mice﻿, patients with a high ratio of intra-tumoral CD8^+^ T lymphocytes with activated CTLs to Tregs were associated with a more favorable clinical outcome in both hepatocellular carcinoma and ovarian cancer^26, 27^. It is worth noting that we found db/db mice had a lower basal CD8^+^ cells than control db/m+ littermates, suggesting their lower basal immunity against tumor. Db/db mice which carry a mutation in the leptin receptor gene are characterized by a complex syndrome with hormonal imbalance, abnormal reproductive function, altered hematopoietic and immune function. In particular, it has been shown that leptin could promote the responsiveness of naive T-cells and favor a shift to a predominantly Th1-type response^28^. Leptin mutation in db/db mice could therefore lead to reduced T-cell function. In addition, it has been shown that hyperglycemia in STZ-induced T1D model may reduce the function of T cells^29^. It is likely that db/db mice which develop hyperglycemia have a similar reduced function of T cells and thus lead to a lower basal CD8^+^ T cells compared with db/m+ control mice as observed in our study.

Similar immunomodulatory effects of exendin-4 have been reported in recent experimental studies. For instances, exendin-4 treatment showed anti-atherosclerotic effect which seems to be partly mediated by suppressing the inflammatory response in activated macrophages characterized by decreased level of tumor necrosis factor-α (TNF-α) and monocyte chemoattractant protein-1 (MCP-1)/CCL2 in activated macrophages^11^. In addition, our group has reported increased inflammatory changes in peripheral blood mononuclear cells (PBMC) collected from patients with T2D compared to control subjects which was reduced by *in vitro* treatment with exendin-4^15^. Similar *in vivo* results have also been reported in T2D patients treated with GLP-1 agonist^30^ although long-term randomized clinical trials using incretin-based therapies (GLP-1 agonist or DPP4-inhibitors) have reported neutral effects^31, 32^.

We hypothesized that up-regulation of inflammatory cytokines upon exendin-4 treatment are mediated through different signaling pathways based on our previous findings and studies from other groups. We have previously shown that increased pro-inflammatory cytokines (such as tumor necrosis factor-α, interleukin-1β, interleukin-6) were attenuated by exendin-4, possibly by suppressing MAPK signaling pathways^15^. In addition, it has been shown that the anti-inflammatory action of exendin-4 might act through STAT-1 and PKA/AKT pathways^33, 34^. Whether up-regulation of inflammatory cytokines of exendin-4 is also mediated through these signaling pathways awaits further confirmation.

The model we used in this study comprised highly immunogenic tumors (virus-induced tumor model), and it is unclear whether exendin-4 would have the same effect on less immunogenic tumors. While our results might need independent replication in other systems, the novel anti-tumor effects of exendin-4 mediated through modulating host immune responses are encouraging. These results further support our hypothesis that exendin-4 may act directly on the immune cell populations known to express GLP-1 receptors and highlight the divergent effects of exendin-4 on tumor biology.

Taken together, our results showed that exendin-4 or possibly other treatment, which could attenuate Tregs and stimulate tumor-specific effector T cells could prevent or control tumor development. These results suggested that exendin-4 might have the potential for cancer immunotherapy, especially for T2D patients although clinical studies will be needed to confirm such notion.

## Conclusions

We have demonstrated exendin-4, besides its effect on lowering BG, possess an ability to suppress tumor growth by improving the CTL/Treg ratio to a higher level. Our findings highlight the complexity of diabetes and cancer with dysregulation of tumor immunity playing a pivotal role in tumor development. While further mechanistic and clinical studies are needed to confirm these findings, our results highlight the importance of identifying aetiological factors and pathological pathways in order to personalize treatment for maximal benefits and minimal harm.

## Materials and Methods

### CUP-1 cells

A murine cell line, namely Chinese University Papillomavirus-1 (CUP-1) was previously established in our laboratory through co-transfection of HPV-16 E7 and H-ras into baby mouse kidney cells harvested from C57BL/KSJ mouse, which shares the same genetic background as db/db mouse^35^. CUP-1 cells were immortalized and exhibited tumorigenic property in nude mice, with continuous expression of functional HPV-16 E7^16^. CUP-1 is tumorigenic in syngeneic db/db mice and allows the study of exendin-4 effect in an immuno-competent environment.

### Mice

C57BL/KSJ-+Leprdb/+Leprdb (db/db) diabetic mice (9–10 weeks) and their littermate control (db/m+) mice were purchased from the Jackson laboratory (MA, USA). Animals were housed under specific pathogen-free conditions at the Laboratory Animal Service Centre (LASEC) according to protocols and guidelines approved by the Animal Experimentation Ethics Committee (AEEC) in The Chinese University of Hong Kong.

### Experimental regime

CUP-1 cells (2 × 10^7^ cells/150 μl PBS) were injected subcutaneously underneath the scruff of 10-week old male db/db mice and db/m+ mice on day 0. We mixed the cell suspension thoroughly every time and injected with 25G syringe slowly. We have confirmed the injected cells were in good morphology by microscopy examination, which means the procedure did not damage the cells. The mice were then randomly assigned into three experimental groups to receive intraperitoneal (ip) administration of exendin-4 at 10 nmol/kg (low dose), or 30 nmol/kg (high dose), or PBS (control) daily for 13 consecutive days (n = 10 for each group). The experimental regime was shown in Fig. 2A.

Body weight, food consumption and random blood glucose (RBG) of the mice were measured every other day during the treatment period. Random blood glucose was measured using glucometer (OneTouch, Canada). Any signs of abnormal symptoms including ulceration or infection at inoculation sites, distress or pain were monitored. After completion of the 13-day exendin-4 treatment, mice were fasted overnight and euthanized with pentobarbitone. Blood was collected from orbital sinus and centrifuged at 3,000 rpm for 15 minutes at 4 °C. Serum was extracted for quantitating insulin levels. CUP-1 tumors were excised for histologic examination.

### Tumor growth assay

Tumor sizes were measured by caliper (Mitutoyo, Taiwan) every other day starting from day 3 of the treatment period. Tumor volume was calculated using the equation V = (a × b^2^) × 0.5236, where “a” represents the larger dimension and “b” is the perpendicular diameter^36^.

### Oral glucose tolerance test (OGTT)

Oral glucose tolerance test (OGTT) was performed on day 9 of the treatment period. Mice were fasted overnight, and glucose solution was administered orally at a dose of 2 g/kg body weight. Blood glucose levels were then determined by performing tail bleeds at 0, 30, 60, 90, and 120 min following glucose challenge using Autokit Glucose (Wako) according to the manufacturer’s procedures. The blood glucose excursion profile from 0 to 120 min was used to calculate the area under the curve (AUC 0–2 h). Serum insulin concentration was measured simultaneously at each indicated time point using the mouse insulin ELISA kit (Millipore, USA).

### Murine splenocyte preparation

Freshly prepared spleens were immediately placed in 5 ml of cold PBS, pressed through a mesh filter, and washed twice with PBS. Erythrocytes were lysed and cells were washed twice with PBS before resuspension in Roswell Park Memorial Institute (RPMI)-1640 Medium (10% FBS, pH7.4).

### Immunofluorescence analysis

The snap frozen tumor tissue were sectioned (thickness: 4  μm) and stained with rat anti-mouse CD8 antibody (1:200) from Abcam (Cambridge, UK). Tissue slides were blocked with 0.1% bovine serum albumin for 1 hour before incubation with primary antibodies overnight at 4 °C. After incubation, the slides were washed 3 times with PBS and then incubated with fluorochrome conjugated secondary antibodies (1:400) for one hour at room temperature. Stained slides were washed 3 times with PBS and mounted with ProLong® Antifade Mountant solution containing 4’, 6-diamidino-2-phenylindole (DAPI) (Invitrogen, Carlsbad, CA). All stained slides were stored in dark at 4 °C, and examined within 3 days using a Zeiss Axioplan 2 imaging microscope (Carl Zeiss, Hamburg, Germany). Representative images were captured using a Spot digital camera (Diagnostic Instruments Inc., Sterling Heights, Michigan, USA). The original magnifications were ×200.

### Cytotoxicity assay

Mice were sacrificed on day 13 of exendin-4 treatment. Their spleens were removed and splenocytes were isolated. Red blood cells were lysed and washed away. The splenocytes were cultured in complete RPMI-1640 medium supplemented with 10% FBS, phorbol 12-myristate 13-acetate (PMA) (10 ng/ml) and E7^49–57^ peptides (synthesized by Synpeptide Co. Ltd, Shanghai, China) for 5 days. The cells were used as effector cells in the CTL assay using CytoTox96 Non-radioactive Cytotoxicity Assay (Promega). CUP-1 cells which expressed HPV-16 E7 were used as target cells. In brief, target cells and effector cells were re-suspended in RPMI-1640 medium. Target cells (1 × 10^4^ cells) were co-cultured with effector cells in different ratios in a 96-well round bottom culture plates at 37 °C. After 4 hours of incubation, the culture plates were centrifuged and the supernatant (50 μl per well) was collected to assess the amount of lactate dehydrogenase (LDH). The percentage of lysis was calculated from the following equation: 100 × (A-B)/(C-D), where A is the reading of experimental-effector signal value, B is the effector spontaneous background signal value, C is maximum signal value from target cells, and D is the target spontaneous background signal value.

### Intracellular cytokine staining and flow cytometric analysis

Splenocytes were harvested on day 13. Before intracellular cytokine staining, splenocytes from each treatment group were incubated for 16 hours with 5 μg/ml E7^49–57^ peptides, containing MHC class I epitope for detecting E7 specific CD8^+^ T cell precursors in the presence of GolgiPlug (1 ul/ml) for detecting E7-specific CD8^+^ T cell precursors. The stimulated splenocytes were then washed twice with FACScan buffer and stained to determine the number of IFN-γ secreting CD8^+^ T cells and CD4^+^CD25^+^FoxP3^+^ Tregs. Fluorescein peridinin chlorophyll protein (PerCP)-conjugated anti-CD4 antibody and allophycocyanin (APC)-conjugated anti-CD25 antibody (Biolegend, San Diego, CA, USA) were used for T cell surface staining, and Alexa Fluor 488-conjugated anti-FoxP3 antibody (BD Pharmingen Corp., San Diego, CA, USA) were used for intracellular staining of the T lymphocyte subpopulation. IFN-γ secreting CD8^+^ T cells and Tregs were gated from total lymphocytes using flow cytometry. Analysis was performed on a Becton-Dickinson FACSCalibur with CELLQuest software (Becton-Dickinson Immunocytometry System, Mountain View, CA USA).

### Splenic cell culture supernatant concentration of cytokines

Splenic cell culture supernatant was harvested and stored at −80 °C for detection of cytokines using the Milliplex MAP kit assay (Merck Millipore, Billerica, MA, USA) with the Bio-Plex 200 suspension array system (BioRad Laboratories, Hercules, CA, USA).

### Statistical analysis

Animal data were expressed as mean ± SD or mean ± SEM. Differences between the groups were examined for statistical significance using one-way ANOVA, followed by Dunnett’s post tests or t test as appropriate. A value of p ≤ 0.05 was considered as statistically significant.

### Ethic approval and consent to participate

The animal study was approved by the Animal Research Ethics Committee of Hong Kong and performed in accordance to the Animals (Control of Experiments) Ordinance (approval No.13/039/MIS).

## Electronic supplementary material

Supporting Information
