# Supplementary material for: Exendin-4 Exhibits Enhanced Anti-tumor Effects in Diabetic Mice
Source: Sci Rep. 2017 May 11;7:1791. doi: 10.1038/s41598-017-01952-5 (PMC5431757; doi:10.1038/s41598-017-01952-5)
Supplement: Supplementary file 1 — Supporting Information [file 41598_2017_1952_MOESM1_ESM.pdf]

Exendin-4 Exhibits Enhanced Anti-tumor Effects in Diabetic Mice

Lan He[1,2,3], Priscilla TY Law[1], Chun Kwok Wong[4,5], Juliana CN Chan[2,3,6], Paul KS Chan[1]

[1]Department of Microbiology, [2]Department of Medicine and Therapeutics, [3]Li Ka Shing Institute of Health Sciences, [4]Department of Chemical Pathology, [5]Institute of Chinese Medicine and State Key Laboratory of Phytochemistry and Plant Resources in West China, The Chinese University of Hong Kong, Hong Kong, [6]Hong Kong Institute of Diabetes and Obesity, The Chinese University of Hong Kong, Prince of Wales Hospital, Shatin, NT Hong Kong

Corresponding to:

Professor Paul KS Chan
Telephone: (852) 2632 3339
Fax: (852) 2632 3426
Email: paulkschan@cuhk.edu.hk
Department of Microbiology,
The Chinese University of Hong Kong
Prince of Wales Hospital, Shatin, Hong Kong

Running title: anti-tumor effect of exendin-4

Keywords: Anti-tumor immune response; CD8+ cytotoxic T cells; T regulatory cells, Diabetes, Tumor, Human papilloma virus (HPV)

Supporting Information

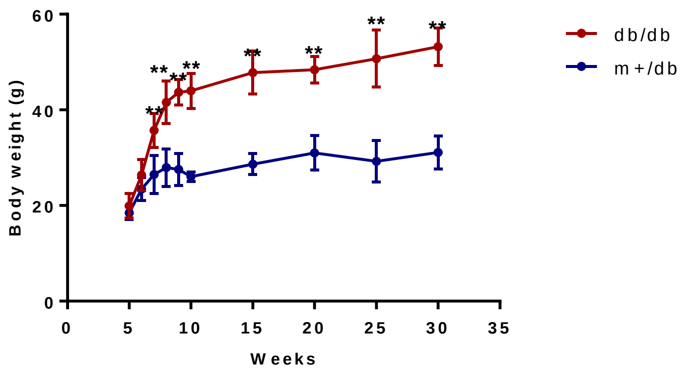

Figure S1. Change in body weight with age in db/db and m+/db (control) mice. Body weight of db/db and non-diabetic littermates (m+/db) was measured weekly from 5 to 30 weeks of age (n=10). Each point represents means ± S.D. ** $p < 0.001$, diabetic group compared with control group.

A. **Fasting serum glucose concentration of db/db and m+/db mice**

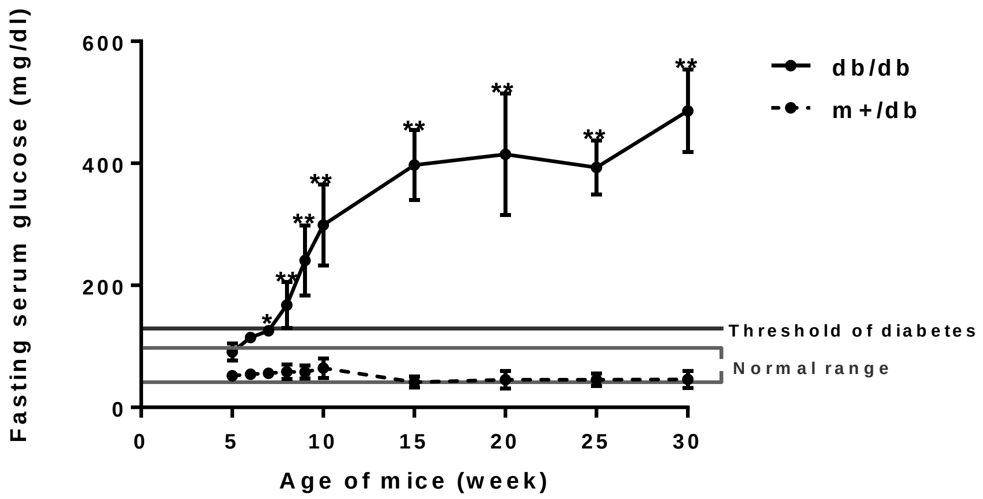

B. **Serum glucose concentration at 2 hours during OGTT of db/db and m+/db mice**

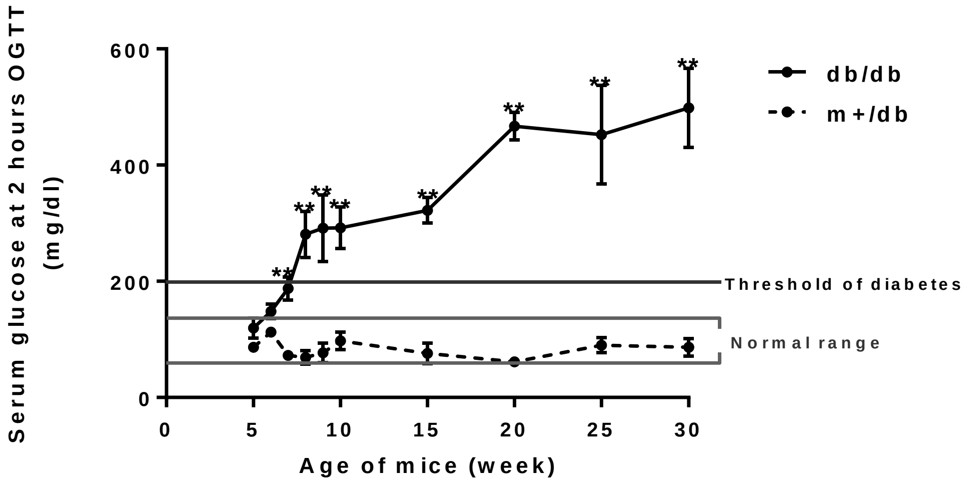

Figure S2. Change in blood glucose level with age in db/db and m+/db mice. Oral glucose tolerance test (OGTT) was performed weekly in db/db and m+/db mice from 5 to 30 weeks of age (n=10). Serum glucose concentration was measured using Autokit Glucose (Wako, Japan). (A) Fasting serum glucose concentration and (B) Serum glucose concentration at 2 hours during OGTT of db/db and m+/db mice are shown. The solid line indicates the threshold for diagnosis of diabetes. Each point represents mean ± S.E.M. * $P < 0.05$, ** $P < 0.001$, db/db compared with m+/db mice

A

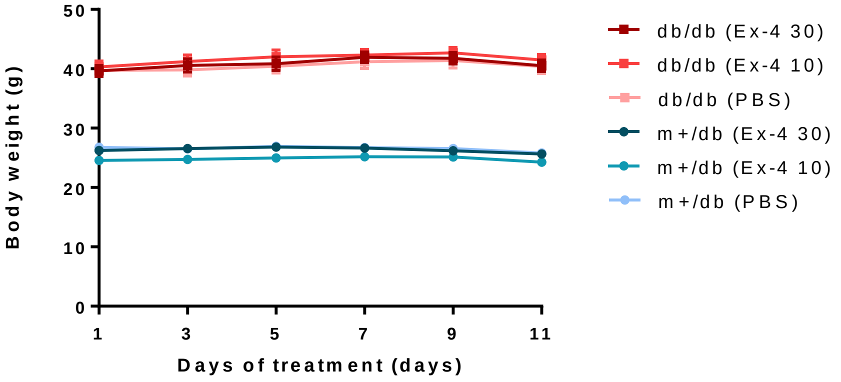

B

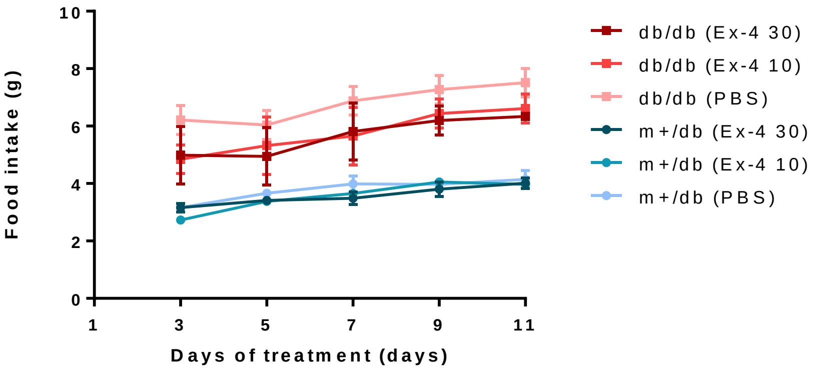

Figure S3. Body weight and daily food consumption in CUP-1 subcutaneous allograft db/db and m+/db mice injection with exendin-4 or PBS (control). Male db/db and db/m+ mice inoculated with 2x10^{7} CUP-1 cells were injected with exendin-4 intraperitoneally (10 or 30 nM/kg body weight) or PBS daily for 13 days. (A) Body weight and (B) daily food consumption were measured every other day in the db/db and m+/db mice (n = 10). Data are expressed as mean ± S.E.M. Ex4 30 indicates the mice treated with 30 nM/kg body weight Exendin-4; Ex4 10 indicates the mice treated with 10 nM/kg body weight Exendin-4.

Parameter	db/db			db/m+		
	PBS	Ex-4 10	Ex-4 30	PBS	Ex-4 10	Ex-4 30
Fasting insulin (ng/ml)[a]	1.7 ± 0.6	2.5 ± 0.6*	2.6 ± 0.6*	0.3 ± 0.1	0.3 ± 0.1	0.2 ± 0.1
HOMA-IR[b]	18.3 ± 0.5	12.1 ± 1.3**	8.5 ± 1.3**	0.9 ± 0.2	1.2 ± 0.3	0.7 ± 0.1
HOMA-IS[b]	65.5 ±8.2	113.4 ± 69.7**	127.1 ± 50.4**	26.8 ± 9.96	28.5 ± 2.38	20.4 ± 3.36

Table S1 Fasting serum insulin, HOMA-IR and HOMA-IS of db/db and m+/db mice in 13-day exendin-4 treatment

Ex-4 10 and Ex-4 30 represent mice treated with 10 and 30 nM/kg exendin-4, respectively. PBS indicates the mice injected with PBS as control. Data are expressed as mean ± S.E.M. * $P < 0.05$, ** $P < 0.001$, compared with PBS in the corresponding db/db or m+/db group.

[a] Fasting serum insulin level was measured at day 13 of exendin-4 treatment using insulin ELISA Kit (Millipore, USA).

[b] Homeostasis model assessment of insulin resistance (HOMA-IR) and insulin secretion (HOMA-IS) were calculated to measure insulin resistance and insulin secretion, respectively.
